# BDNF Polymorphisms Are Linked to Poorer Working Memory Performance, Reduced Cerebellar and Hippocampal Volumes and Differences in Prefrontal Cortex in a Swedish Elderly Population

**DOI:** 10.1371/journal.pone.0082707

**Published:** 2014-01-23

**Authors:** Samantha J. Brooks, Emil K. Nilsson, Josefin A. Jacobsson, Dan J. Stein, Robert Fredriksson, Lars Lind, Helgi B. Schiöth

**Affiliations:** 1 Department of Neuroscience, Functional Pharmacology, Uppsala University, Uppsala, Sweden; 2 Department of Medical Sciences, Uppsala University, Uppsala, Sweden; 3 Department of Psychiatry and Mental Health, Faculty of Health Sciences, University of Cape Town, Observatory, Cape Town, South Africa; University of Electronic Science and Technology of China, China

## Abstract

**Background:**

Brain-derived neurotrophic factor (BDNF) links learning, memory and cognitive decline in elderly, but evidence linking BDNF allele variation, cognition and brain structural differences is lacking.

**Methods:**

367 elderly Swedish men (n = 181) and women (n = 186) from Prospective Investigation of the Vasculature in Uppsala seniors (PIVUS) were genotyped and the BDNF functional rs6265 SNP was further examined in subjects who completed the Trail Making Task (TMT), verbal fluency task, and had a magnetic resonance imaging (MRI) scan. Voxel-based morphometry (VBM) examined brain structure, cognition and links with BDNF.

**Results:**

The functional BDNF SNP (rs6265,) predicted better working memory performance on the TMT with positive association of the Met rs6265, and was linked with greater cerebellar, precuneus, left superior frontal gyrus and bilateral hippocampal volume, and reduced brainstem and bilateral posterior cingulate volumes.

**Conclusions:**

The functional BDNF polymorphism influences brain volume in regions associated with memory and regulation of sensorimotor control, with the Met rs6265 allele potentially being more beneficial to these functions in the elderly.

## Introduction

Brain derived neurotrophic factor (BDNF) plays a crucial role in activity-dependent brain plasticity [1], [2] especially in relation to learning and memory [3], [4] such that long term potentiation and memory consolidation purportedly declines as we age [5]. One variant, rs6265, also known as Val66Met, is the frequently studied functional single nucleotide polymorphism (SNP) in the BDNF gene at 11p13, resulting in a valine/methionine substitution. The Val allele of this SNP has been linked to exercise-related memory enhancement in humans [6]. In a similar vein, exercise-induced memory enhancement in rats is linked to BDNF signalling in the hippocampus [7], [8] and in the striatum [9]. Adults carrying the Met allele have associated smaller brain activation in mesolimbic regions, and hippocampal function (e.g. long term potential and memory consolidation) is strongly associated with cellular mechanisms that are mediated by BDNF [5]. Additionally, increased Body Mass Index (BMI) in children and adults is associated with BDNF [10], [11] and excessive food intake may significantly reduce the production of BDNF, leading to detrimental effects on cognition [12]. In addition, healthy young Chinese adults carrying the homozygous Met allele have smaller brain volume in frontal, temporal, cingulate and insula cortices [13] and Japanese Met carriers, particularly females, have age-related atrophy in the bilateral dorsolateral prefrontal cortex [14]. Moreover, in two separate experiments in young and old cohorts, it has been shown that elderly Met/Met carriers, in comparison to a younger cohort, recall fewer items on an associative memory test than Val carriers [15]. This suggests that the genetic effects on cognition are more pronounced as cognitive resources diminish during the ageing process, particularly for those carrying the Met allele. However, elderly people have also been shown to have better Stroop performance and enhanced brain activation when carrying the Met allele [16] and recent evidence links the homozygous Val allele of the Val66Met BDNF gene to deficits in working memory processes in healthy elderly [17]. Thus, the data linking BDNF on brain structure and function in elderly is inconsistent.

In this study, we focus on the commonly studied functional rs6265 in relation to brain volume and cognition. Three additional highly linked BDNF SNPs were genotyped (7103411, 7124442 and 2049045). Variants in rs7103411 and rs6265 have been linked to poorer cognitive performance on a battery of neuropsychological tests, particularly in memory for rs7103411 and for the met allele on rs6265 in elderly females, although perceptual speed for rs6265 met was shown to be significantly better than for Val carriers [18]. Furthermore, rs7124442 is associated with the development of anorexia and bulimia nervosa [19], both weight-related psychiatric diseases also characterized by cognitive dysfunction. The fourth SNP rs2049045 has not been extensively studied. However, we did not include these in our analyses with brain volume and cognitive function. We decided only to examine the rs6265 SNP because it is the only functional polymorphism of the 4 genotyped, it has been extensively studied in other populations, and was highly linked to the other three SNPs in our cohortthe three non-functional SNPs were highly linked in our cohort to the functional polymorphism rs6265.. Against this background, here we aim to examine, for the first time, the functional BDNF SNP in our cohort, and its relation to memory-related cognition and brain structure in an elderly population of Swedish men and women from the Prospective Investigation of Vasculature in Uppsala Seniors (PIVUS) study [20]. We hypothesise that variations on the functional BDNF allele in an elderly population will be associated with poorer cognitive function and structural brain differences in global brain volumes, and regions associated with memory (e.g. hippocampus, prefrontal cortex).

## Methods

### Subjects

A total of 367 elderly Swedish men (n = 181) and women (n = 186) between 70 and 75 years of age, who had had a brain scan, from the Prospective Investigation of the Vasculature in Uppsala seniors (PIVUS) [20] were included in this study, which was conducted in Uppsala, Sweden. The study was approved by the Ethics Committee of the University of Uppsala and all participants gave written informed consent. The participants included in this study were a subset chosen from the total PIVUS cohort of 1016 people [20] based on several inclusion/exclusion criteria described further below. From the original 1016, 409 participants had participated in a brain scan. Of these 409, 27 had either suffered a stroke between the ages 70 and 75, or there were indications of cortical infarcts, and were excluded. 2 subjects who had a Mini Mental State Score (MMSE) of 24 or below at the time of the MRI scan were excluded. Of the remaining 380 cognitively healthy subjects, 13 were excluded due to missing genotype data, leaving a total of 367 subjects available for further analyses. The maximum time the subjects could score on the TMT-B was 600 seconds. Subjects who reached this score were not included in analysis because a ceiling effect would weaken the analysis of variance. Finally, 12 subjects lacked TMT-B data, thus 345 subjects was used. See Table 1 for demographics and genetic data of included subjects, and Table S1 for PRISMA flowchart of exclusion/inclusions.

**Table 1:** Demographics of participants' distribution on the three genotypes of 6265.

Independent of SNP			rs6265		
	Male	Female	AA	AG	GG
Male/Female	186	181	4/8	46/51	136/122
BMI	26.7 (3.8)	26.7 (4.6)	29.2(5.8)	26.8 (4.3)	26.6 (4.1)
Trailmaking-task B	156 (92)	150 (71)	118 (44)	143 (62)	160 (90)
Educational level Scale 1–3	0.6 (0.8)	0.7 (0.8)	0.9 (0.9)	0.6 (0.9)	0.6 (0.8)
Total brain volume (ml)	1819 (141)	1659 (144)	1778 (149)	1731 (166)	1742 (163)
Total grey matter (ml)	600 (68)	534 (61)	565 (62)	562 (66)	569 (75)
Gray matter relative TBV (%)	33 (3.3)	32 (3.3)	34 (3.4)	32 (2.9)	33 (3.5)
White matter (ml)	469 (68)	416 (51)	402 (44)	447 (55)	443 (70)
White matter relative TBV (%)	26 (3.9)	25 (2.3)	24 (2.5)	27 (2.3)	25 (3.5)
CSF (ml)	750 (100)	710 (104)	751 (86)	717 (106)	734 (104)
CSF relative TBV (%)	41 (3.9)	43 (4.2)	42 (3.6)	41 (3.8)	42 (4.2)
Grey matter+White matter (ml)	1006 (113)	1014 (112)	1006 (78)	1007 (113)	1012 (114)

Unless otherwise stated. Mean values are given with standard deviations in parentheses.

### Cognitive measures

#### Trail Making Test

The TMT is a test of various aspects of executive function (e.g. mental speed, divided attention and working memory) with varying difficulty [i.e. TMT version A (TMT-A) and TMT version B (TMT-B)], which is associated with frontal lobe and hippocampal function [21]. During the TMT-A, participants are required to ‘join the dots’ representing 25 targets of ascending numbers (1-2-3-4-5…) on a sheet of paper. For the TMT-B, participants respond in the same manner, but instead alternate between a set of numbers (1-13) and a set of letters (A-L), again in ascending order (1-A-2-B-3-C-4-D-5…). In essence the TMT-B task incorporates working memory and set-shifting capabilities, as participants are required to follow mentally the progression of two consecutive yet differing lists. The participants must start their trial on the circle marked ‘Begin’, and continue until they reach the endpoint, a circle marked ‘End’. The goal is to complete the task as quickly as possible, and the outcome measurement is time in seconds.

#### Verbal fluency test

Performance on the verbal fluency test required participants to recall verbally and spontaneously as many different animal names as possible within 60 seconds. The time it took to give responses was recorded, with repeated answers discounted from the total score.

### Covariates

In all assessments we used Body Mass Index (BMI), total matter (gray+white) volume (TMV), gender and education attainment as covariates, due their potential confounding effects on brain structure and function. We chose to include both gray and white matter volume because we are examining both volume and function (e.g. in terms of Trail Making performance), and gray matter is inextricably linked to white matter. For example, previous research links weight to brain structure, e.g. [12], and to worse working memory performance and smaller brain volumes in the elderly [22]. Furthermore, global and regional brain volume reduction is commonly observed in the elderly [23]. Females have also been shown to have lower brain volumes than males [24]. However, we chose to exclude cerebrospinal fluid (CSF), as there is evidence that in some cases, the measurement of CSF is most susceptible to error in SPM [25]. Finally, education level was used as a covariate, given that it can impact on brain volume, particularly the hippocampus, and cognition in the elderly [26]. The educational level (basic level = 1, college = 2, university = 3) for each subject was assessed by means of a standardized questionnaire.

### Genotyping and linkage disequilibrium analysis

Four BDNF SNPs were genotyped: the functional rs6265 (position: chr11:27, 636, 492), rs7103411 (position: chr11: 27,656,701), rs7124442 (position: chr11: 27,633,617) and rs2049045 (position: chr11:27, 694,241). However, linkage disequilibrium analysis revealed high similarity between the four genotypes (D′>0.9) and thus only the functional snp was subjected to further analysis in our cohort. We focused on the functional 6265 polymorphism, given that it has been extensively studied in other cohorts previously, and also because it is the functional variant of this gene, and thus perhaps more relevant to brain function. For rs6265, the A allele encodes for methionine (Met) and the G allele encodes for valine (Val). The A allele is less frequent in rs6265, making methionine the uncommon amino acid at position 66 of the BDNF protein. Genetic information is found in Table S1. The SNPs genotyped in our cohort have previously been associated with Body Mass Index (BMI) and cognition in elderly populations [10], [11], [15], [18]. BMI was considered a confound, given previous research on its link to brain structure, e.g. [12], and used as a regressor in our analyses. Furthermore, we have previously shown in the same population, that being overweight is linked to worse working memory performance and smaller brain volumes [22]. Linkage information was calculated using R statistical computing in with the “genetics” package installed [27] and is found in Table S2.

Genotyping of the four SNPs was carried out at the SNP technology platform at Uppsala University (www.genotyping.se/) using an Illumina Golden Gate Assay (Illumina Inc., San Diego, CA, USA). Genotyping was conducted by investigators who were blind to the purpose of the study, and deviance from the Hardy-Weinberg equilibrium was assessed using a chi-squared test, and rs6265 can be considered to be in equilibrium (p<0.05)..

### MRI acquisition

Global and regional measures of brain volume were acquired with magnetic resonance imaging (MRI) and processed using voxel based morphometry (VBM) in SPM8 (http://www.fil.ion.ucl.ac.uk/spm/software/spm8/), a technique that uses an algorithm to determine local concentrations of gray matter (GM), white matter (WM) and cerebral spinal fluid (CSF) densities on a voxel by voxel basis [28]. A high resolution three-dimensional T1-weighted volumetric turbo field echo scan was acquired using a Philips 1.5 Tesla scanner (Gyroscan NT, Philips Medical Systems, Best, The Netherlands). The three dimensional gradient echo sequence was used with scan parameters: repetition time (TR) 8.6 ms, echo time (TE) 4.0 ms, and flip angle 8 degrees. Sagittal slices with a field of view of 240 mm, a slice thickness of 1.2 mm, inter-slice interval of 0.9 mm and an in-slice resolution of 0.94×0.94 were reconstructed.

### MRI processing

Morphological changes in GM were calculated using SPM8, by segmenting GM from white matter and cerebrospinal fluid using the unified segmentation approach [29]. Following the segmentation procedure, probability maps of GM were ‘modulated’ in order to control for the effect of spatial normalization by multiplying the probability value of each voxel by its relative volume in native space before and after warping. We then normalized GM images, based on probability maps, into Montreal Neurological Institute (MNI) standard space for further contrast analyses in an additive model for each allele. Modulated images were smoothed with an 8-mm full width half maximum (FWHM) Gaussian kernel, in line with recent VBM studies by our group and others [22], [30]. This smoothing kernel was applied prior to statistical analyses to increase the signal to noise and to correct for image variability.

### Statistical analyses

Statistical analysis was conducted on the functional BDNF SNP (rs6265) and its relationship to the dependent variables (TMT-B, verbal fluency) was done using R [27]. We controlled for BMI as weight may be a significant confound on cognition, particularly hippocampal-related memory performance [5], [12]. Furthermore, sex may significantly alter the size and structure of the brain [31] particularly in the hippocampus and other subcortical brain regions, and particularly in the elderly [15]. Additionally, level of education attainment during adolescence may impact on cerebral volume [32]. Thus it was important to identify weight, sex, education and total matter volume (TMV) as confounding variables.

### VBM analyses

All VBM analyses were carried out using the SPM8 VBM GUI function (VBM 8.1 version 1.20; www.fil.ion.ucl.ac.uk/spm). Our VBM analyses consisted first of contrasts between any A allele (Met) versus G (Val), and second, of linear regressions examining the association between genotype and brain volumes: all analyses corrected for the confounding variables. We chose to conduct both a contrast analysis and regression analyses, so that we could test both a dominant model and an additive model. The contrast analyses allow for the testing of a dominant model, that is, comparing any A allele (the presence of any Met allele) with the G allele (the presence of a Val only allele). However, with the dominant model approach we may fail to observe a more discrete linear relationship between gene and brain volume (with covariates) that is better examined with an additive model (accounting for 3 types of genotype: AA, AG, GG). We also conducted a post-hoc region of interest regression analysis in the bilateral hippocampus, due to its association with BDNF and memory e.g. [5]. We selected the hippocampus mask using the PickAtlas anatomical labeling (aal) toolbox function in SPM8 (http://fmri.wfubmc.edu/software/PickAtlas), in order to avoid the problem of ‘double-dipping’. We ran a contrast analysis between any A allele (Met) versus G allele (Val) using the hippocampus mask, correcting for BMI, sex and total matter volume (TMV) by adding these variables as regressors. Subsequently we conducted a regression analysis with the hippocampus mask, using TMT-B score as the dependent variable, again correcting for BMI, sex and total matter volume (TMV). Unless otherwise stated, the activations we report are at this exploratory stage are uncorrected.

## Results

### Participant demographics

The A (Met) allele in rs6265 was slightly more common in females (67%). However, we demonstrate no significant gender differences (independent of SNP variation) between BMI, trail making B score, education and total matter volume.. We correct all subsequent analyses for gender (as well as BMI, total matter volume and education attainment). See Table 1.

### Association between SNPs and cognitive scores (Verbal fluency and TMT-B score)

**Figure 1:**
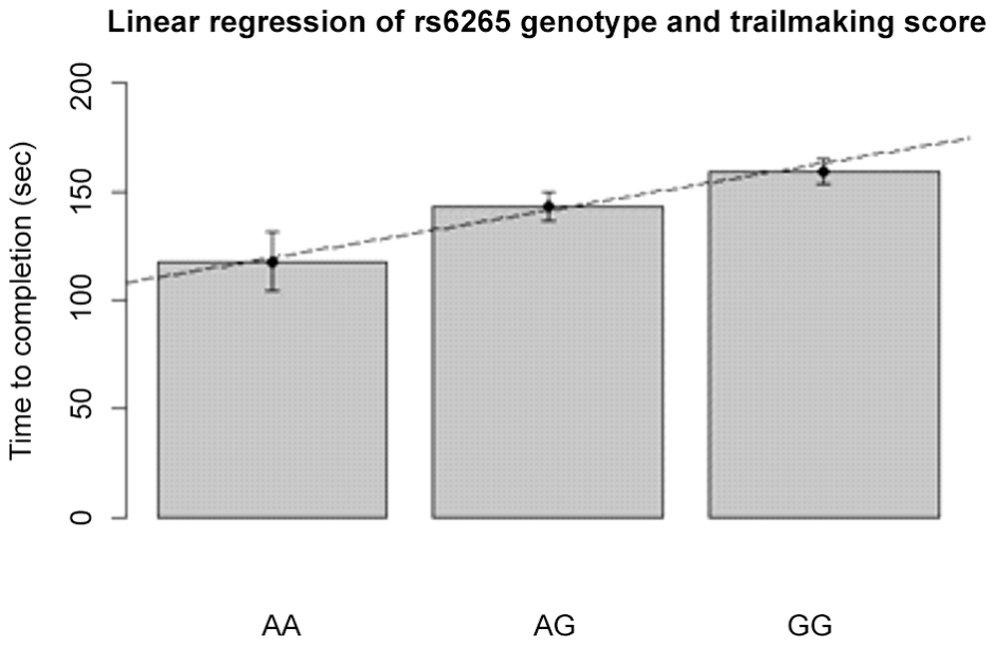
Linear regression in rs6265 (Val66Met) with Trail Making Task B performance. Error bars represent standard error (for slope and p values see table 2).

We examined associations separately between rs6265and scores on the Trail Making Task B (TMT-B) and Verbal Fluency task. Covariates to correct for potential confounds were total matter volume (TMV), BMI, sex and education level. Linear models investigating association with verbal fluency did not yield significant results. The BDNF 6265 SNP showed an uncorrected significant association with the TMT-B scores as shown in Table 2. Figure 1 depicts the association between TMT-B and the most commonly studied and functional rs6265 SNP.

**Table 2:** Results from linear regression analysis and snp information of the analyzed snp's.

Rs number	Risk allele	Pvalue	Slope (s/ra)	Slope std	Chisq 367	Chisq 345
6265	G	0.0147	19.76	8.05	0.59(0.45)	0.23(0.69)

Slope is given in seconds per risk allele. Trailmaking regression is for a subset of 345 subjects due to 22 subjects either having missing or ceiling Trailmaking scores. Values in parenthesis are simulated P-values based on 1000 replicates.

### VBM results

#### Whole Brain Contrast analysis in rs6265

**Figure 2:**
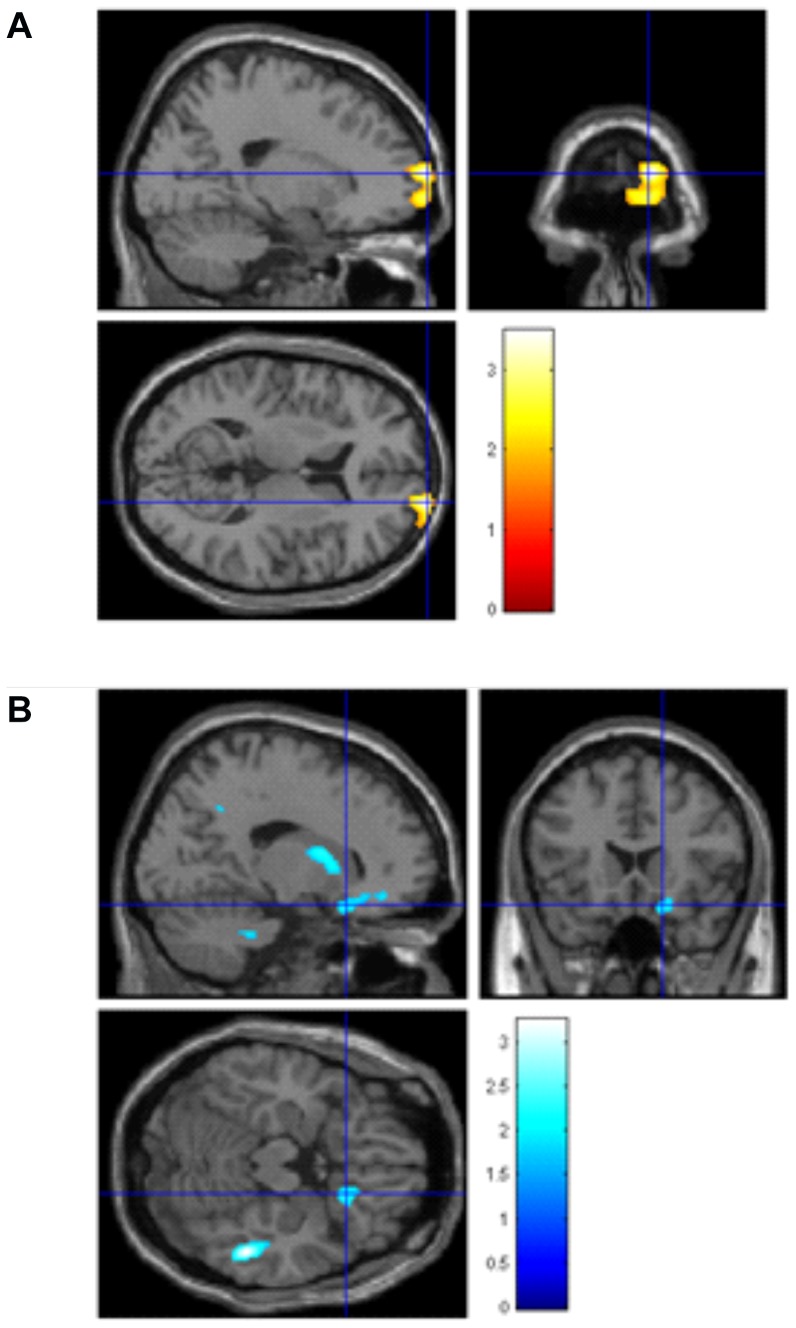
VBM Contrast analyses in the rs6265 SNP A/AG versus GG allele. Left side: Larger brain volumes in right medial prefrontal cortex (x = 20, y = 74, z = 10, p = 0.002). Right side: Smaller brain volume in the right occipito-temporal gyrus, x = 52, y = −38, z = −18, p = 0.04) and right orbitofrontal cortex x = 18, y = 18, z = −18); T bars represent volume difference as T-statistic.

See Fig. 2, Table 3. By contrasting those with any A, representative of the met allele (e.g. AA, AG) versus no A, for rs6265, and correcting for matter volume, sex, education and BMI, we found that those with any A (Met) allele had increased volume in the right medial prefrontal cortex (MNI coordinates x = 20, y = 74, z = 10,t = 3.49, p<0.001) and right caudate body (MNI coordinates x = 6, y = 16, z = 4,t = 2.74, p = 0.001), and decreased volume in the right occipito-temporal gyrus (MNI coordinates x = 52, y = −38, z = −16, t = 3.22, p = 0.001) and right orbitofrontal cortex (MNI coordinates x = 18, y = 20, z = −18, t = 2.31, p = 0.01).

**Table 3:** Contrast, regression and region of interest analyses in voxel-based morphometry (VBM); all analyses corrected for age, gender, brain matter volume (gray+white matter) and highest level of education achieved.

Brain Regions	BA	Laterality	x	y	z	T value	Cluster size	P value
**Contrast AA [met] versus GG rs6265 (** **Fig. 2** **.)**								
Increased medial PFC	10	Right	20	74	10	3.49	N/A	<0.001*
Increased caudate body	–	Right	6	16	4	2.75	N/A	0.001*
Decreased occipito-temporal gyrus	37	Right	52	−38	−16	3.22	N/A	0.001*
Decreased OFC	47	Right	18	20	−18	2.31	N/A	0.01*
**Whole brain regression analysis in rs6265 (correlation with A [met] allele) (** **Fig. 3** **.)**								
Increased cerebellum	–	Right	58	−64	−38	3.74	3077	0.01**
Increased cerebellum	–	Left	−45	−50	−32	3.12	3080	0.01**
Decreased thalamus	–	Right	12	−8	−4	3.14	N/A	0.001*
**Whole brain regression analysis showing negative (faster TMT-B, larger brain volume) or positive (slower TMT-B, larger brain volume) association with TMT-B scores (** **Fig. 4** **)**								
Negative cerebellum	–	Left	−38	−36	−34	4.16	N/A	<0.001*
Negative precuneus	7	Right	26	−52	72	3.32	2456	0.03**
Negative superior frontal gyrus	8	Left	−16	30	56	3.84	53984	0.008***
Positive brainstem/pons	–	Left	−12	−30	−40	3.95	1789	0.05**
Positive posterior cingulate	31	Left	−22	−44	46	3.07	N/A	0.001*
Positive posterior cingulate	31	Right	34	−60	22	3.03	N/A	0.001*
**Region of interest regression analysis in hippocampus in rs6265 (positive correlation with any A [met] allele)**								
Hippocampus	35	Left	−24	−14	−16	1.68	N/A	0.02*
**Region of interest regression analysis showing negative association with TMT-B scores and brain volume (** **Fig. 5** **.)**								
Hippocampus	35	Left	−16	−4	−22	3.52	N/A	<0.001*
Hippocampus	–	Right	32	−12	−24	3.13	N/A	0.001*

Contrast between AA/AG vs. GG rs6265; Whole brain regression analysis in allele variation rs6265(AA = 1, AG = 2, GG = 3); Whole brain regression analysis in relation to TMT-B scores; Region of interest regression analysis using hippocampus mask (PickAtlas aal); Region of interest regression analysis using hippocampus mask.

BA = Brodmann's Area; MNI Coordinates = x, Saggital plane, y, Coronal plane, z, Axial plane; p* = uncorrected peak level, p**uncorrected cluster level, p***FWE corrected cluster level.

#### Whole Brain Linear Regression analyses in rs6265

**Figure 3:**
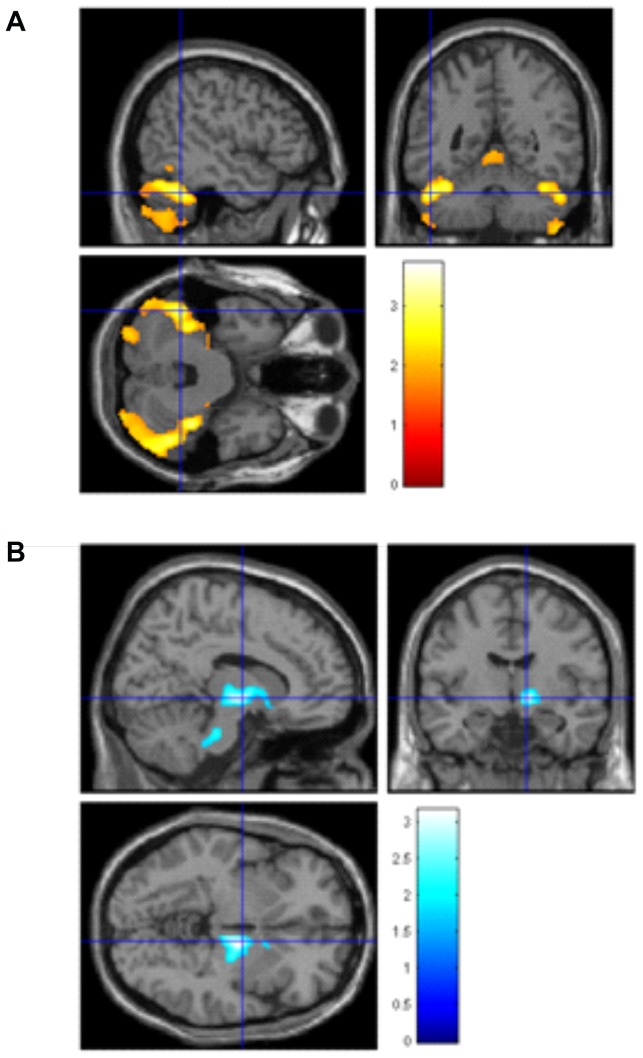
VBM regression analyses in the rs6265 SNP. Left side: Larger brain volume in the bilateral cerebellum in the AA/AG versus GG allele, x = 58/−45, y = −64/−50, z = −38/−32; Right side: Smaller volume in the right thalamus/brainstem in AA/AG versus GG allele, x = 12, y = −8, z = −4. T bars represent volume difference as T-statistic.

See Fig. 3, Table 3. In our first linear regression model, with total matter volume, sex BMI and education as covariates we examined how allele frequency for rs6265 was associated with brain volumes. We found greater bilateral cerebellar volume that most significantly regressed with the A (met) allele of rs6265 (MNI coordinates x = 58/−45, y = −64/−50, z = −38/−32, t = 3.74/3.12, p = 0.01/p = 0.01). Additionally, we found a FDR corrected peak level smaller brain volume in the right thalamus/brainstem (MNI coordinates x = 12, y = −8, z = −4, t = 3.14, p = 0.001) associated most strongly with the A (met) allele.

**Figure 4:**
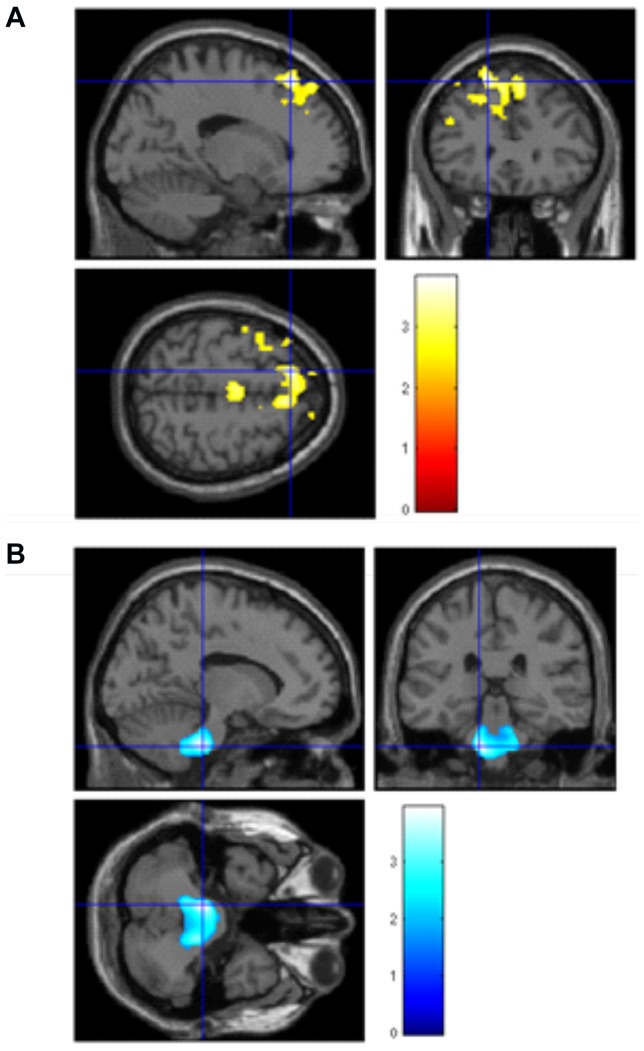
Regression analysis independent of SNP allele status, showing how faster working memory performance on the TMT-B task is associated with, left side:larger brain volume in the left superior frontal gyrus (x = −16, y = 30, z = 56, right side: smaller brain volume in the brainstem/pons (saggital x = −12, coronal y = −30, axial z = −40). T bars represent volume difference as T-statistic.

See Fig. 4 Table 3. In a second linear regression model independent of allele status, but this time to examine the association between TMT-B (working memory) scores and brain volume, we again used matter volume, BMI, education and sex as covariates of no interest. We found negative associations (the lower, and thus better the TMT-B scores, the larger the brain volume in the left cerebellum (MNI coordinates x = −38, y = −36, z = −34,t = 4.16, p<0.001), right precuneus (MNI coordinates x = 26, y = −52, z = 72, t = 3.32, p = 0.03), and left superior frontal gyrus (MNI coordinates x = −16, y = 30, z = 56, t = 3.84, p = 0.008). We found positive associations (the higher, and thus worse the TMT-B scores, the larger the brain volume) in the left brainstem/pons (MNI coordinates x = −12, y = −30, z = −40, t = 3.95, p = 0.05) and bilateral posterior cingulate cortex (MNI coordinates x = −22, y = −44, z = 46,t = 3.07, p = 0.001/x = 34, y = −60, z = 22, t = 3.03, p = 0.001). As an additional step we re-ran the analyses to include allele code for rs6265 but this did not alter the results observed.

#### Region of interest (ROI) Linear Regression analyses in rs6265

Finally, given the known relationship between hippocampus, BDNF expression and memory function e.g. [5], we conducted post-hoc regression analyses, correcting for total matter volume, BMI, sex and education, with a region of interest (ROI) mask for the bilateral hippocampus to examine specifically the association with rs6265. As a first step with the ROI mask, we found a positive association in line with the A (met) allele and greater brain volume in the left hippocampus (MNI coordinates x = −24, y = −14, z = −16, t = 1.68, p = 0.02). In other words, those with the any A (met) allele had larger left hippocampal volume.

**Figure 5:**
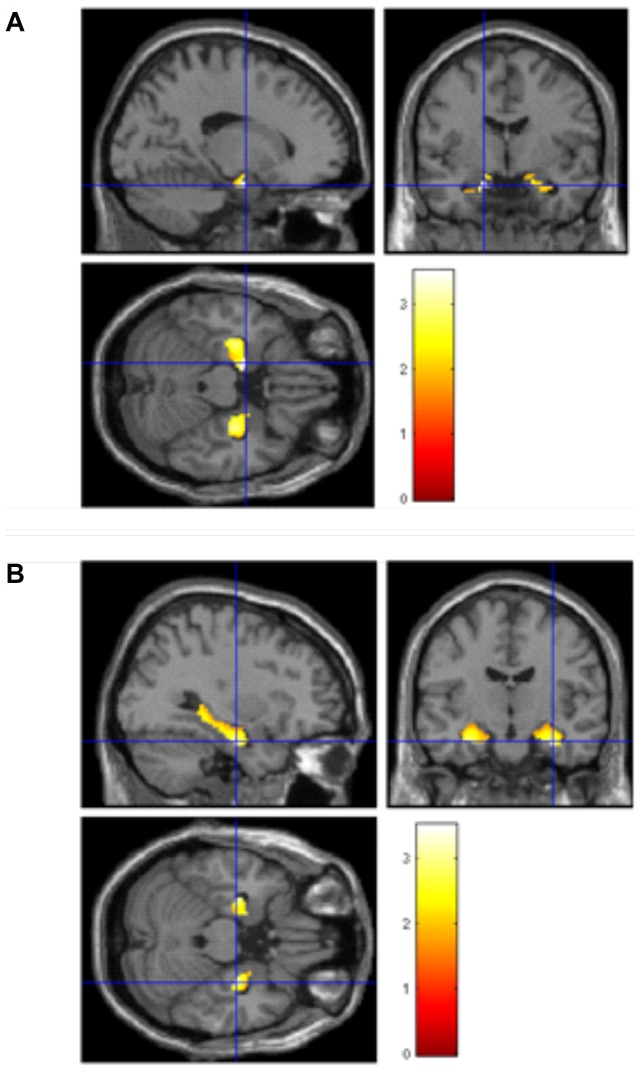
Region of interest regression analysis with age, sex and total matter volume as covariates, showing greater bilateral hippocampal volume is associated with faster TMT-B scores. Left side: Left hippocampus saggital x = −16, coronal y = −4, axial z = −22; Right side: Right hippocampus saggital x = 32, coronal y = −12, axial z = −24. T bars represent volume difference as T-statistic.

As a second step, to examine the association between TMT-B performance and hippocampal volume, we ran a second regression using the same covariates and found a negative correlation (the lower, thus better the TMT-B score, the larger the brain volume) in bilateral hippocampus (MNI coordinates x = −16, y = −4, z = −22, t = 3.52, p<0.001; x = 32, y = −12, z = −24, t = 3.13, p = 0.001). See Fig. 5, table 3. As a final step, to link allele variation status, TMT-B scores and brain volume in the hippocampi, we re-ran the above masked regression analysis but this time included a regressor to denote allele code (e.g. AA = 1, AG = 2, GG = 3). There was no significant correlation found for rs6265.

## Discussion

We show that the risk allele in the functional rs6265 (Val66Met) BDNF polymorphism is associated with deficits in memory-related cognition (working memory), and differences in regional brain volumes in an elderly population of Swedish men and women. We found that the any A allele (Met) on rs6265 is linked to faster working memory performance in this elderly population. Additionally, in an additive model regression analysis we showed that those elderly people possessing the seemingly beneficial any A (Met) allele on rs6265 had larger brain volumes in the bilateral hippocampus andbilateral cerebellum, and reduced volume in the right thalamus. In a dominant model contrast analysis, we found significantly larger volumes in the right caudate body, right medial prefrontal cortex (regions linked to memory, motor coordination and decision making), and smaller volumes in the right occipito-temporal lobe and right thalamus (regions linked to somatosensory responses and arousal). Furthermore, greater bilateral hippocampal, cerebellum and precuneus, with smaller brainstem and bilateral posterior cingulate were associated with better working memory performance independent of allele variation, in an additive model. No association was found between the 6265 BDNF functional SNP and verbal fluency performance or with Body Mass Index (BMI) in our elderly cohort, and no differences were found in global brain volumes. Thus, our data suggests that variation on the BDNF gene, specifically the rs6265 SNP contribute to cognitive and anatomical variation, and that the Met allele may be more beneficial to cognitive function in the elderly. Collectively, our data suggest that larger brain volumes in memory, decision-making and motor regions, combined with smaller volumes in somatosensory/arousal regions may indicate mediation effects by BDNF leading to greater cognitive control and less arousal interference (e.g. via peripheral autonomic responses) in elderly people.

We examined the relationship between rs6265 and brain volumes using both additive (regression) and dominant (contrast) analyses, so that we could explore linear relationships, as well as extreme contrasts in brain volume between the allele variations on this gene. Consequently, we find different, though not contradictory results following these analyses, demonstrating that brain volume changes in some regions are more susceptible to the additive model, which are not observed in the more extreme dominant model. The dominant contrast model shows that prefrontal, occipito-temporal and caudate regions are altered by the presence of any Met allele, whereas the bilateral cerebellum and thalamus show differences when measuring the presence of none, 1 or 2 copies of the Met allele, in a more discrete additive regression model. Given the link between higher order learning, memory and the 6265 BDNF SNP (e.g. Mattson et al., 2004), our dominant contrast analyses were only able to show differences in brain regions associated with higher order learning and memory (e.g. PFC, occipito-temporal gyrus, caudate body), which may be the strongest link to the BDNF gene in terms of brain function. Whereas the additive regression models in our analyses showed links between the 6265 SNP and regions associated with motor coordination, arousal and emotion recognition systems (bilateral cerebellum, brainstem, precuneus and bilateral posterior cingulate), arguably regions that are more prone to individual variation, particularly in an ageing population with vast life experience differences.

We found both larger and smaller gray matter volume in the rs6265 BDNF polymorphism, which may have functional significance. Specifically, greater brain volumes were found in relation to those with the Met allele, in the right medial prefrontal cortex, right caudate and bilateral cerebellum. Additionally, better working memory task performance in those with the Met allele was associated with larger left cerebellum, right precuneus and bilateral hippocampal volumes. These brain regions are perhaps collectively involved in better memory function, particularly in those elderly people who have the Met allele (especially the homozygous variant). Previous research has linked cognitive function in older adults to declining volume in the cortico-cerebellar pathways [33], and so having at least one copy of the Met BDNF allele may help to preserve these brain regions from age-related atrophy and cognitive decline. Although it must be noted that the Met allele of the 6265 BDNF SNP is associated with worse cognitive function [34]. However, there is also some evidence that the 6265 BDNF Met allele influences hippocampal glutamatergic activity [35], which may positively influence memory in older adults. Thus, the effects on brain structure and function of having at least one, if not two copies of the Met allele in older age, is still not yet clear. Smaller brain volumes in those with the Met allele were also observed in our older cohort, in the right occipito-temporal gyrus, right OFC, and right thalamus, which may be associated with reduced function in attention and arousal brain networks, especially in relation to emotional processing [36]. Perhaps those older adults carrying the Met allele are less aroused by emotional stimuli, which ordinarily interferes with memory processing. Consistent with this notion, better working memory performance was associated with reduced volumes in the brainstem and bilateral posterior cingulate, regions also associated with emotional arousal.

BDNF regulates neuronal growth and brain plasticity in memory-related brain structures, such as the hippocampus, but the beneficial effects of BDNF on learning and memory seem to diminish with age [5]. Our data suggest that poorer performance on a working memory task and related brain differences are specifically associated with the Val allele rs6265 BDNF allele in an elderly Swedish cohort, and that carrying the Met allele may have neuro-protective effects. This supports recent findings linking the homozygous Val allele of the Val66Met BDNF gene to deficits in working memory processes in healthy elderly [17]. Furthermore, elderly Met carriers have been shown to be better at the Stroop task and show better brain function during tasks as measured by event related potentials [16]. Our study expands on these findings by measuring brain volume in the elderly.

However, ours and the data described above contradict previous reports that it is Met, not Val allele carriers who have poorer performance on a cognitive task and alterations in brain volume. Thus, genetic variation effects on brain structure and function (particularly memory) are obviously complex, and likely also involve the effects of many other factors, such as age, life stressors, diet, exercise etc. For example, in rs6265, young adults carrying the Met allele have deficits in brain function [5], young Met carriers have less exercise-related memory enhancement [6] and elderly Met carriers have been shown to recall fewer items during a memory test than Val carriers, a difference that was not observed in a younger cohort [15]. Furthermore, healthy young Chinese adults carrying the Met allele have smaller brain volumes in frontal, temporal, cingulate and insula cortices [13]. Thus, it could be that the beneficial effects of carrying the Met rs6265 allele are more apparent in the elderly than in younger persons, or that other environmental factors play a large part.

We found that those elderly people in our Swedish cohort carrying the Val allele, who were slower during the working memory task, had smaller right and left cerebellum volumes, caudate body and right medial prefrontal cortex, as well as larger thalamus, occipito-temporal and orbitofrontal volumes. Smaller cerebellar volume likely entails less efficient motor responses during this paper-based working memory task requiring motor dexterity, and indeed the cerebellum is involved in motor coordination, specifically sensorimotor control [37], with greater volumes linked to better performance on a working memory task [38]. In support of this interpretation, Laing and colleagues reported that elderly carriers of the Val rs6265 allele had slower perceptual speed during paper-based memory tasks [18] but they did not also measure brain volume in the elderly as we did.

The caudate body is also involved in motor coordination, and is one of the main regions of atrophy underlying degenerative motor impairment [39]. In a similar vein, smaller caudate volume in our Val elderly carriers predicted slower motor responses on the working memory task, thus BDNF-related deficits may specifically target dopaminergic neurons in the caudate in the elderly, leading to motor impairment. Furthermore, age-related alterations in connectivity between medial prefrontal and caudate regions are linked to memory impairments [40], suggesting that reduced medial prefrontal cortex observed in our Val carriers also contributes to working memory deficits.

Larger thalamus volume may indicate plasticity due to greater sensory neural traffic, which in turn might interfere with cerebellar motor coordination, given the connectivity between these brain regions [41]. Thus, smaller thalamus and greater cerebellar, caudate and medial prefrontal volumes in elderly persons carrying the Met allele may underlie better sensorimotor control and enhanced working memory performance, as seen in our cohort.

We also observed that those elderly people carrying the rs6265 Val allele, who had worse performance on the working memory task, had larger occipito-temporal and orbitofrontal volumes. The occipito-temporal cortex is part of the ventral visual processing stream, serving recognition, visual thought, planning and memory (Milner, 2012), whereas the orbitofrontal cortex is involved in decision making [42]. Greater volume in these regions may be indicative of greater effort to sustain effective cognitive performance in the elderly, especially if increased plasticity in regions underpinning peripheral autonomic arousal (e.g. heart rate, perspiration, muscle contraction) leads to interference effects on focused attention, such that heightened arousal may complicate decision making processes.

In a similar vein, larger bilateral posterior cingulate cortex volume was associated with worse working memory performance in our cohort, a brain region involved in the default mode network, e.g. a network in which function is increased during idle periods, but reduced during focused attention [43]. Functional decreases in the posterior cingulate cortex are associated with better cognitive performance following cognitive training [44]. Thus, it might be, if we consider our other volumetric findings, reduced arousal responses in the brains of elderly subjects (e.g. heart rate, perspiration, muscle contraction) are linked to better cognitive and sensorimotor control. Reduced plasticity in midbrain and default mode network (e.g., thalamus, posterior cingulate cortex), but greater plasticity in cerebellum, medial prefrontal cortex and caudate, which are supported by BDNF expression, may reduce arousal interference and strengthen cognitive control. Elderly carriers of the Met allele may have hypoactivation in mesolimbic regions, as a consequence of reduced plasticity, which might be detrimental to motivated cognitive performance in the young. From this perspective, it might be beneficial for elderly, less agile subjects, to rely on cognitive, rather than arousal brain systems, which might explain the switch in the beneficial effects of the Met SNP between young and old.

Faster working memory performance correlated with greater brain volume in the bilateral hippocampus in our elderly cohort but perhaps most significantly on the left side. This compliments previous extensive research that BDNF regulates brain plasticity and memory function, particularly in relation to the hippocampus e.g. [5], [7], [8]. But to our knowledge, we are the first to demonstrate a link between hippocampus size and working memory performance in an elderly cohort, whose enhanced working memory performance was also associated with the Met allele of the rs6265 BDNF SNP.

### Strengths and limitations

The main strength of this study is that the sample studied here was on a homogenous sample of elderly Swedish men and women from one town in Sweden. Thus, our cohort would have had similar educational and life experiences, which might otherwise lead to major confounds in a less homogenous population. We also took measures to ensure that our cohort were otherwise healthy in terms of cognitive ability and neuroanatomical infarcts. Furthermore, we corrected for sex, Body Mass Index, education and total matter volume which are all factors that may influence brain function and structure. Furthermore, unlike many other studies into BDNF in humans, we examined four highly linked BDNF SNPs and their influence on two cognitive measures and brain volume. However, we were only able to report uncorrected data in all but one cluster, which for an exploratory study such as ours is acceptable, but does highlight that greater statistical power is needed to detect more robust findings. Future VBM studies that can report FWE- or FDR-corrected clusters, to correct for multiple comparisons and to rule out spurious findings, will progress this work further. Additionally, due to a lack of longitudinal data, we cannot speculate on whether a specific BDNF SNP is more or less detrimental to cognitive function and brain structure in the elderly compared to younger populations. Finally, we included only measures of verbal fluency, mental speed, divided attention and working memory (using verbal fluency and Trail Making tasks), thus preventing an exploration into how variation on the BDNF allele might influence other cognitive functions, which could be explored further, by adding additional neuropsychological tasks in future studies.

In summary, our data suggest an association between BDNF rs6265 (val66met) and working memory ability in an elderly population of Swedish men and women. Furthermore, greater brain volume was associated with the Met allele of rs6265 in the right medial prefrontal cortex, cerebellum, caudate body and hippocampi (regions linked to memory, motor coordination and decision making), as well as smaller volume in the right occipito-temporal gyrus and thalamus (regions linked to somatosensory responses and arousal). Additionally, larger bilateral hippocampal, cerebellum and precuneus, with smaller brainstem and bilateral posterior cingulate cortex were associated with better working memory performance independent of allele variation. Thus, the Met allele of rs6265 appears to be more beneficial to the elderly than the Val allele, in terms of brain health and memory function. Given their role, these brain regions suggest that the Met allele of BDNF promotes plasticity in functions associated with an ability to exert control over one's sensorimotor processes. The link to brain volume and working memory performance was independent of BDNF polymorphisms, and therefore it seems likely that other environmental factors are at play. Nevertheless, susceptibility to develop memory deficits in later life may be exacerbated by one's genetic predisposition. Given that exercise is linked to greater levels of BDNF in the brain, adopting healthier lifestyle choices, e.g. increasing one's daily exercise regime, may help to counter some of these detrimental cognitive effects, particularly in elderly persons.

## Supporting Information

Figure S1 **PRISMA Diagram.**
(DOC)

Table S1 **Descriptive statistics of linked SNPs.**
(DOCX)

Table S2 **Linkage and distance information for all possible combinations of the four BDNF SNPs included.**
(DOCX)
