# Supplementary material for: BDNF Polymorphisms Are Linked to Poorer Working Memory Performance, Reduced Cerebellar and Hippocampal Volumes and Differences in Prefrontal Cortex in a Swedish Elderly Population
Source: PLoS One. 2014 Jan 23;9(1):e82707. doi: 10.1371/journal.pone.0082707 (PMC3900399; doi:10.1371/journal.pone.0082707)
Supplement: Figure S1 — PRISMA Diagram. (DOC) [file pone.0082707.s001.doc]

**Identification**

**Screening**

**Eligibility**

**Included**

1016 Initial Subjects

409 Had MRI data

382 scanned non-strokers

Two subjects had MMSE scores of 24 or below

380 Cognitively healthy scanned syubjects

27 Had a stroke between 70 and 75

13 was missing genotype data

Study population of 367 for genetic analysis, 380 for other analysis.
