# Supplementary material for: BDNF Polymorphisms Are Linked to Poorer Working Memory Performance, Reduced Cerebellar and Hippocampal Volumes and Differences in Prefrontal Cortex in a Swedish Elderly Population
Source: PLoS One. 2014 Jan 23;9(1):e82707. doi: 10.1371/journal.pone.0082707 (PMC3900399; doi:10.1371/journal.pone.0082707)
Supplement: Table S1 — Descriptive statistics of linked SNPs. (DOCX) [file pone.0082707.s002.docx]

| Rs number | MAF % | Minor allele | Location | Snp type |
| --- | --- | --- | --- | --- |
| 6265 | 16 | A | 11:27679916 | G/A |
| 7124442 | 35 | G | 11:27677041 | G/A |
| 2049045 | 16 | C | 11:27694241 | C/G |
| 7103411 | 19 | G | 11:27700125 | G/A |

Supplementary table 1: Descriptive statistics of linked snp's
