# Supplementary material for: BDNF Polymorphisms Are Linked to Poorer Working Memory Performance, Reduced Cerebellar and Hippocampal Volumes and Differences in Prefrontal Cortex in a Swedish Elderly Population
Source: PLoS One. 2014 Jan 23;9(1):e82707. doi: 10.1371/journal.pone.0082707 (PMC3900399; doi:10.1371/journal.pone.0082707)
Supplement: Table S2 — Linkage and distance information for all possible combinations of the four BDNF SNPs included. (DOCX) [file pone.0082707.s003.docx]

| Rs number | Rs number | D | D' | Corr. Coef. | Distance (bp) |
| --- | --- | --- | --- | --- | --- |
| 7124442 | 6265 | 0,134 | 0,999 | -0,326 | 2875 |
| 7124442 | 2049045 | -0,057 | 0,999 | -0,324 | 17200 |
| 7124442 | 7103411 | -0,065 | 0,999 | -0,350 | 23084 |
| 6265 | 2049045 | 0,136 | 0,999 | 0,995 | 14325 |
| 6265 | 7103411 | 0,065 | 0,999 | 0,931 | 20209 |
| 2049045 | 7103411 | 0,133 | 0,999 | 0,927 | 5884 |

Supplementary table 2:Linkage and distance information for all possible combinations of the four BDNF snp's included.
